# Prevalence and risk factors of inadequate medicine home storage: a community-based study

**DOI:** 10.11606/S1518-8787.2017051000053

**Published:** 2017-11-07

**Authors:** Rand Randall Martins, Andreza Duarte Farias, Yonara Monique da Costa Oliveira, Rodrigo dos Santos Diniz, Antonio Gouveia Oliveira

**Affiliations:** I Universidade Federal do Rio Grande do Norte. Faculdade de Farmácia. Departamento de Farmácia. Natal, RN, Brasil; II Universidade Federal de Campina Grande. Unidade de Saúde, Centro de Educação e Saúde. Cuité, PB, Brasil

**Keywords:** Patient Medication Knowledge, Health Knowledge, Attitudes, Practice, Drug Utilization, Drug Storage, Patient Education as Topic, Drug Information Services

## Abstract

**OBJECTIVE:**

Assess the extent of inadequate home storage of medicines andidentify important risk factors.

**METHODS:**

A cross-sectional survey based on a probability sample in the community, conducted in 267 households in Cuité, State of Paraíba, Northeast Brazil, in 2014. Logistic regression was used to study the risk factors.

**RESULTS:**

The prevalence of households with inadequate storage was 76.0%. Problems with storage include direct exposure to sunlight in 10.9% of households, the presence of dust in 23.6%, and storage within reach of children in 76.0%. Medications no longer used are usually disposed of into the environment in 92.1% of households. Inadequate storage is more likely when home organization of medications is the responsibility of a male subject (OR = 1.729) or an older person (OR = 1.029), when out of date medicines are found (OR = 2.963), and in households with no children (OR = 2.088).

**CONCLUSIONS:**

Physicians and pharmacists should advise patients on how to adequately store medicines at home, especially when the person in charge of medications is a male or an older adult, and if there are no children in the household.

## INTRODUCTION

Home storage of medications is a common practice around the world and includes both prescription medicines and over-the-counter (OTC) medications indicated for acute and chronic conditions^1^ ^,^ ^2^. In recent years, medicine consumption patterns have changed, resulting in larger purchasing volumes and, consequently, excessive amounts stored at home^1^.

In addition to the higher costs^2^, the accumulation of medications at home may be associated with harm to the patients, to their family, and even to the environment. An association between the accumulation of medications and multiple storage locations at home, and unfavorable clinical outcomes or decreased adherence to treatment has been described^3^. This negative association is more likely to be harmful to older adults, who frequently have multiple diseases and a greater number of medications at home, leading to a higher frequency of administration errors, medication interactions, and adverse reactions^4^. A large quantity of medicines in the household has also been implicated in an increased risk of inappropriate self-medication, especially through the administrations of leftover medicines, including nonsteroidal anti-inflammatory medications, corticosteroids, opioids, and antimicrobials^1^. Regarding the latter medications, they are often found in households, representing a potential public health problem due to the risk of the development of multidrug-resistant organisms^5^ tahnks to improper disposal. In many countries, non-existent public regulations for the proper disposal of medications results in the widespread use of the domestic waste and the public sewage system for that purpose, leading to extensive environmental contamination^6^ ^,^ ^7^. Consequently, it is important to conduct research within the community to understand the prevailing practices of home storage and disposal of medicinal products. This knowledge is important to health professionals, as it may provide guidance for their advice to patients regarding adequate home storage and proper disposal of medications, decreasing the untoward accumulation of medications in the household and its consequent non-rational use. In addition to the risks related to the accumulation of medications already mentioned, the inadequate storage of medications can cause changes in their pharmacological characteristics^6^ ^,^ ^8^ ^,^ ^9^ and lead to accidental poisoning^10^ ^,^ ^11^.

However, despite the relevance and the serious implications for public health of this subject, studies addressing this issue are scarce and have adopted heterogeneous methodologies and definitions. The problem is complex because the home storage of medicines is influenced by multiple factors, including cultural factors^12^, making it difficult to compare studies originating from different countries. Some studies have investigated the home storage of medications by direct interviews at the time of acquisition in community pharmacies^13^ ^,^ ^14^ or via telephone surveys^15^ ^,^ ^16^. Other studies have analyzed medicines collected during campaigns for medications disposal to infer how the medications had been previously stored at home^6^. These approaches have the drawback of failing to observe the actual storage locations and the involved medications, in addition to being susceptible to recall bias and the underreporting of sensitive issues, such as storage of medications within reach of children and medications past the expiration date. Studies that directly inspected the storage locations and the types of stored medications often have small sample sizes,^17^ the home visit was previously scheduled, leading to possible interference in the veracity of the interview^1^, or the data analysis was limited to simple descriptions^14,17–20^.

The objectives of the present study were to assess, in a population-based sample and through a household survey with direct inspection of storage locations, the prevalence of medicine home storage, the extent of inadequate home storage, the methods of medicine disposal, and the identification of inadequate storage risk factors.

## METHODS

This cross-sectional, observational survey was conducted in Cuité, a community of about 20,000 inhabitants located in the State of Paraíba, in Northeastern Brazil, between May and June 2014. A stratified proportional random sample was obtained from the 12 census strata in which the town is divided. In each stratum, one street was randomly selected and the house with the lowest home address number was the first to be visited. From there, the home visits followed a strict order: the second house from the left, then the house across the street. This sequence was repeated until the predefined number of adults for that stratum was interviewed.

The studied population consisted of subjects older than 18 years-old, of either gender, who were the persons in charge for the acquisition and storage of medicines in each household. The interview was conducted by a trained and supervised member of staff, and the data were recorded in structured data collection forms. The subjects who declined to allow observation of the medications storage site were not included.

The subjects were questioned regarding age, gender, income, education, number of residents in the household, and number of children under 12 years old. During the interview, medication storage locations were directly inspected and all the medications found in the household were recorded. The storage location was characterized with respect to which room of the house it was located in, the temperature in Fahrenheit, relative humidity, the presence of pollutants (food particles and other organic materials), height from the ground (being considered out of reach of children when above 170 cm), and whether the medications were kept in a container with a lid used exclusively for medicines (“home pharmacy”). Data collection was always performed at the same time of the day, between 3 p.m. and 6 p.m. Home storage was considered inadequate when at least one of these characteristics was observed: medications within reach of children, medicines not stored in recipients meant exclusively for this purpose, the presence of pollutants, and humidity. Regulatory agencies and medical societies are emphatic regarding the need to keep medicines out of reach of children and locked in cabinets where medications can be organized and protected from heat, humidity, light, and dust^22^ ^,^ ^23^. In our study, we followed these guidelines to define the criteria of adequate medicine home storage. The medications were classified by Anatomical Therapeutic Chemical Code (ATC) and pharmaceutical form and checked for the expiration date, utilization in the previous seven days, and presence of proper packaging. The usual method of medication disposal in the household was also recorded. The pilot study was conducted in 10 households located in a different region of the main data collection. During the data collection in the household, interviewers were supervised by the authors. With the pilot study, it was possible to detect problems in the questionnaire, calculate the time of the interview and evaluate the performance of interviewers. However, the validation of the data collection tool was not performed.

A sample size of 270 households was calculated to afford a maximum estimate error of six percentage points with 95% confidence intervals. Statistical analysis was performed using Stata software release 12 (Stata Corporation, College Station, TX, USA). For the descriptive analysis, demographic, clinical, and economic variables were presented as absolute and relative frequencies or as means and standard deviations, as appropriate. For population estimates, exact (binomial) confidence intervals are presented. Univariate logistic regression analyses were performed to identify variables associated with inappropriate medication storage, and odds ratios and their 95% confidence intervals are reported. Variables associated with inappropriate medication storage at the p < 0.10 significance level in the univariate analysis were included in a multivariate logistic regression model and significant associations were considered if p < 0.05.

The study was approved by the Research Ethics Committee of the Hospital Universitário Alcides Carneiro, Universidade Federal de Campina Grande (CAAE 03361212.2.0000.5182).

## RESULTS

In this study, we evaluated 267 households. The mean age of those responsible for medication acquisition and storage was 54.9 (SD = 19.2) years, 190 (71.2%) of them were females and most (62.2%) had only elementary school education or less. There was an average of 3.3 (SD = 1.4) residents per household, 35.2% had children, and the monthly income per household was 1.7 (SD = 0.9) minimum wages (MW).

A total of 803 medications were observed in the 267 households, with an average of 3.7 (SD = 2.1) medications per household. The predominant medicines were cardiovascular medications (28.7%), followed by central nervous system medications (21.8%), and gastrointestinal tract and metabolism medications (16.7%). There was a predominance of solid and semi-solid pharmaceutical forms (77.2%). Most medications had no secondary packaging (66.8%) and 38.4% had not been used in the previous seven days. There were few medicines past their expiration date (19, 2.4%). Table 1 summarizes these data.

**Table 1:** Basic characteristics of subjects, households and stored medicines.

Characteristic	Values	
Person in charge of medicines		
Age in years (mean, SD)	54.9	19.2
Female gender (n, %)	190	71.2
Education > elementary school (n, %)	166	62.2
Household		
Income in MW (mean, SD)	1.7	0.9
Residents per household (mean, SD)	3.3	1.4
Households with children (n, %)	94	35.2
Medicines		
Number of stored medicines (mean, SD)	3.7	2.1
Solid and semi-solid formulations (n, %)	620	77.2
Absence of secondary packaging (n, %)	536	66.8
Not used in the previous week (n, %)	308	8.4
Expired validity (n, %)	19	2.4

MW: minimum wage (R$678.00 equivalent to approximately US$340.00 at the time of data acquisition)

The prevalence of households with inadequate storage was 76.0% (95%CI 70.5–81.0). Direct exposure to sunlight was observed in 29 households (10.9%, 95%CI 7.4–15.2) and the presence of pollutants in 63 (23.6%, 95%CI 18.6–29.1). Medications were often stored within the reach of children, as was observed in 76.0% (95%CI 70.5–81.0) of households. The average temperature and relative humidity in the storage location were 86.7°F (SD = 36.1; equivalent to 30.4°C [SD = 2.3]) and 50.4% (SD = 7.3), respectively. The house room most often used for storage of medicines was the kitchen (52.9%, 95%CI 49.5–56.5), followed by the bedroom (33.2%; 95%CI 30.0–36.7). The most common method of medicine disposal was into the environment, either through the household waste or into the public sewerage system (92.1%, 95%CI 88.2–95.1). Table 2 summarizes these data.

**Table 2:** Characteristics of home storage of medications and methods of disposal.

Characteristic	Values	95%CI
Inadequate storage (n, %)	203 (76.0%)	70.5–81.0
Direct exposure of sunlight (n, %)	29 (10.9%)	7.4–15.2
Contamination with dust (n, %)	63 (23.6%)	18.6–29.1
Within the reach of children (n, %)	203 (76.0%)	70.5–81.0
Room temperature (^o^F, SD)	86.7 (36.1)	
Room temperature (^o^C, SD)	30.4 (2.3)	
Relative humidity (%)	50.4 (7.3)	
Predominant storage room (n, %)		
Kitchen	141 (52.9%)	45.5–57.8
Living room	19 (7.1%)	4.3–10.9
Bedroom	89 (33.2%)	27.7–39.3
Others	18 (6.8%)	4.0–10.5
Medicine disposal (n, %)		
Home garbage or public sewage system	246 (92.1%)	88.2–95.1
Returned to health facilities	21 (7.9%)	4.9–11.8

Univariate logistic regression analyses (Table 3) showed that the inadequate storage of medications is more frequent when the organizer of home medicines is older (OR = 1.037, 95%CI 1.027–1.046), is a male (OR = 1.589, 95%CI 1.024–2.464), and has less education (OR = 2.381, 95%CI 1.715–3.311). Inadequate storage decreases with the presence of children at home (OR = 0.314, 95%CI 0.225–0.439).

**Table 3:** Univariate and multivariate analyses of factors associated with inadequate home storage of medicines.

Characteristic	Univariate		Multivariate	
	
	Odds ratio (95%CI)	p	Odds ratio (95%CI)	p
Person in charge of medicines				
Age in years	1.037 (1.027–1.046)	< 0.001	1.029 (1.016–1.042)	< 0.001
Male gender	1.589 (1.024–2.464)	0.039	1.729 (1.087–2.752)	0.021
Education > elementary school	0.420 (0.302–0.583)	< 0.001	0.998 (0.652–1.529)	0.994
Household				
Income in MW	0.833 (0.693–1.000)	0.051	0.822 (0.664–1.018)	0.073
Residents per household	1.012 (0.960–1.067)	0.656		
Households with children	0.314 (0.225–0.439)	< 0.001	0.479 (0.325–0.706)	< 0.001
Medicines				
Number of stored medicines	0.974 (0.903–1.052)	0.504		
Liquid formulations	0.742 (0.511–1.076)	0.115		
No secondary packaging	1.311 (0.936–1.837)	0.115		
Not used in the previous week	1.325 (0.953–1.840)	0.094	0.813 (0.561–1.180)	0.277
Expired validity	2.355 (0.933–5.942)	0.070	2.963 (1.046–8.397)	0.041

MW: minimum wage (R$678.00 equivalent to approximately US$340.00 at the time of data acquisition)

The results of multiple logistic regression analysis (Table 3) showed that independent factors increasing the risk of inadequate storage are: person responsible for the medications were male (adjusted OR = 1.729, 95%CI 1.087–2.752, p = 0.02), of older age (adjusted OR = 1.029, 95%CI 1.016–1.042, p < 0.001), presence of expired medications in the household (adjusted OR = 2.963, 95%CI 1.046–8.397, p = 0.04), and household with no children (adjusted OR = 2.088, 95%CI 1.416–3.077, p < 0.01). Nearly reaching statistical significance was a lower household income (adjusted OR = 1.217, 95%CI 0.908–1.506, p = 0.073).

## DISCUSSION

This study is relevant to healthcare professionals and to the community in general because it sheds some light into the modalities of home storage of medicinal products and the extent of inadequate storage, and identifies some key factors for an increased risk of inadequate storage. Our main findings were that medications were stored inadequately in the majority of homes, that the most frequent problem was storage of medications within reach of the children, and that the risk of inadequate storage was greater when home medicine organizers were male, of advanced age, and when there are out of date medicines. In contrast, the presence of children at home decreases the risk of inadequate storage.

Increased consumption increases the possibility of accumulating medications at home, both those prescribed for long-term treatment and those prescribed for short time administration^2^. Nevertheless, regardless of the purpose of the treatment, the adequacy of medicine home storage is influenced by the characteristics of the medicines and the characteristics of the subjects. For example, when improperly packaged, pharmaceuticals can lose effectiveness or toxicity can increase^6^. Chemical, physical, and microbiological changes may be caused by extremes of temperature, humidity, and light, especially if the medicinal product is kept out of its original packaging, or stored near food and chemicals^9^. Environmental conditions may also play a key role. Thus, in a first analysis, the correct way to store medications should primarily consider their protection from these environmental factors, in order to maintain their effectiveness and safety. With regard to characteristics related to the subject, accumulation of medications at home favors administration errors and decreases medication adherence, especially in older adults with visual and cognitive impairment^3^. Greater availability of medications at home may promote self-medication by, eventually, promoting the utilization of leftovers from previous treatments and of over-the-counter medicines for treating symptoms that had been previously experienced, thus reducing the search for healthcare consultations^1^ ^,^ ^21^.

The storage location in the household also influences the adequacy of storage. Storing medicines in places easily accessible is a risk factor for the occurrence of accidental poisoning in children and suicide attempts^10^ ^,^ ^11^. Sometimes a solution to one issue has untoward consequences. For example, in Belgium, it is common to use home medication cabinets, which adequately maintain the chemical integrity of medicines, but these may also facilitate the access by children and adolescents^1^. The storage of medicines on top of the kitchen refrigerator is common practice in some Middle Eastern countries, which, while preventing the access of children to medicines, also exposes medications to excess heat generated by the equipment and to soiling during the preparation of food^14^ ^,^ ^16^.

Therefore, notwithstanding the potentially hazardous consequences to individuals, especially children and to patients, and of being a public health problem, there is no consensus on what would be considered adequate home storage of medications. In our study, we followed the guidelines from regulatory agencies to define the criteria of adequate home storage of medications – out of reach of children and protected from heat, humidity, light, and dust^22^ ^,^ ^23^. We also attempted to be systematic and objective in other aspects of the methodology of the study. For example, during the execution of the work, the medications and their storage were directly observed in their actual location at the time of the interview, and the respondent was the family member responsible for keeping and organizing medicines at home.

Inadequate storage was associated with age in our data. Advanced age, with its consequent increase in the prevalence of chronic diseases, is strongly associated with the number of medications administered daily^24^ and the quantities stored at home^3^. Large amounts of medications at home increase the risk of administration errors, adverse reactions, medication interactions^4^, and the use of expired medications. In addition, older people often have sight, motor, and memory limitatios^25^, which may promote the placement of medications in easily accessible places and dispersed inside the house. This disorganization of medications, with multiple storage locations and frequent absence of the packaging and the summary of product characteristics, may cause lower medication adherence^5^.

Our data indicate that the organization of medications by male subjects is strongly associated with inadequate storage. The responsibility for medication management at home falling into women is a common pattern in many parts of the world. In Australia, women tend to buy and store medications at home more often than men^3^. A study in Uganda showed that women are more willing to talk about medicines and are traditionally responsible for the health of children within the family^26^, a similar finding was seen in a Greek study^17^.

A positive aspect revealed in our study is that the presence of children in households is generally associated with greater care in medication storage. Medication stored within reach of children is an important risk factor for serious accidental poisoning^11^. In Turkey, between 1985 and 2008, 65% of cases of domestic poisoning in children was associated with medications^10^. The same study showed that, in 2,251 cases of poisoning in children, 82% was due to easy access to the medication at home. The prevalence of homes with medicines within the reach of children in our sample was high (76%), well above that of United States (30%)^15^, Belgian (33%)^1^, and Serbian (20%) households^19^.

The disposal of medications in the home waste and the sewage system was common. Lack of adequate processing of medication leftovers carries the risk of environmental contamination^15^. In the United States, a significant amount of water sources for human consumption contain traces of medications^7^. This form of disposal is common in many countries^15^ ^,^ ^17^ ^,^ ^19^.

The social and economic characteristics of the population in our study reduce the scope of possible generalizations because the prevalence of inadequate medicine home storage is likely to be closely linked to economic development. For example, in the United State, there is a significant accumulation of medications in households, reflecting excessive consumption made possible by economic wealth^2^. In contrast, in Uganda, after two decades of civil war, only 35% of households store medications^26^. Our study has other limitations. Regional characteristics may make it difficult to generalize the findings to other major cities and beyond the Brazilian Northeast. A consensual definition of adequate medicine home storage is not available, but we tried to overcome this problem by adopting a definition based on the guidelines of international organizations. Nevertheless, methodological aspects of our study that contribute to the validity of the results include being a population study based on a probability sample, data obtained by direct interview without previous appointment, and direct observation of the storage location.

Our results address a subject relevant but poorly understood by health professionals. This survey based on a probability sample of households has shown inadequate storage of medicines in the majority of homes, most often because medications are kept within reach of children. The study identified variables that may signal inadequate home storage of medicines, namely the keeper of medicines at home is male and of older age, the lack of children in the household, and the presence of expired medications. Almost all medications are disposed of through domestic waste or the public sewage system. These results emphasize the need for prescribing physicians and pharmacists to inform patients on how to properly store medications at home, especially those who have any of the risk factors here identified.
